# Medical expenditures associated with nonfatal occupational injuries among immigrant and U.S.-born workers

**DOI:** 10.1186/1471-2458-12-678

**Published:** 2012-08-20

**Authors:** Huiyun Xiang, Junxin Shi, Bo Lu, Krista Wheeler, Weiyan Zhao, J R Wilkins, Gary A Smith

**Affiliations:** 1 Center for Injury Research and Policy, The Research Institute at Nationwide Children’s Hospital, College of Medicine, The Ohio State University, 700 Children’s Drive, Columbus, OH 43205, USA; 2 Division of Biostatistics, College of Public Health, The Ohio State University, Columbus, OH, USA; 3 Division of Epidemiology, College of Public Health, Ohio State University, Columbus, OH, USA

## Abstract

**Background:**

No national study has investigated whether immigrant workers are less likely than U.S.-workers to seek medical treatment after occupational injuries and whether the payment source differs between two groups.

**Methods:**

Using the 2004–2009 Medical Expenditure Panel Survey (MEPS) data, we estimated the annual incidence rate of nonfatal occupational injuries per 100 workers. Logistic regression models were fitted to test whether injured immigrant workers were less likely than U.S.-born workers to seek professional medical treatment after occupational injuries. We also estimated the average mean medical expenditures per injured worker during the 2 year MEPS reference period using linear regression analysis, adjusting for gender, age, race, marital status, education, poverty level, and insurance. Types of service and sources of payment were compared between U.S.-born and immigrant workers.

**Results:**

A total of 1,909 injured U.S.-born workers reported 2,176 occupational injury events and 508 injured immigrant workers reported 560 occupational injury events. The annual nonfatal incidence rate per 100 workers was 4.0% (95% CI: 3.8%-4.3%) for U.S.-born workers and 3.0% (95% CI: 2.6%-3.3%) for immigrant workers. Medical treatment was sought after 77.3% (95% CI: 75.1%-79.4%) of the occupational injuries suffered by U.S.-born workers and 75.6% (95% CI: 69.8%-80.7%) of the occupational injuries suffered by immigrant workers. The average medical expenditure per injured worker in the 2 year MEPS reference period was $2357 for the U.S.-born workers and $2,351 for immigrant workers (in 2009 U.S. dollars, *P* = 0.99). Workers’ compensation paid 57.0% (95% CI: 49.4%-63.6%) of the total expenditures for U.S.-born workers and 43.2% (95% CI: 33.0%-53.7%) for immigrant workers. U.S.-born workers paid 6.7% (95% CI: 5.5%-8.3%) and immigrant workers paid 7.1% (95% CI: 5.2%-9.6%) out-of-pocket.

**Conclusions:**

Immigrant workers had a statistically significant lower incidence rate of nonfatal occupational injuries than U.S.-born workers. There was no significant difference in seeking medical treatment and in the mean expenditures per injured worker between the two groups. The proportion of total expenditures paid by workers’ compensation was smaller (marginally significant) for immigrant workers than for U.S.-born workers.

## Background

According to Bureau of Labor Statistics for the year 2009, the occupational injury and illness incidence rate among private industry employers has declined significantly each year since 2003. The number of private industry nonfatal occupational injuries and illnesses reported in 2009 declined to 3.3 million cases from 4 million cases in 2007 [1]. Public sector data were collected for the first time in 2008, and there were nearly 863,000 injury and illness cases reported among state and local government workers in 2009. Bureau of Labor Statistics estimated that injuries represented 93.6% of nonfatal injuries and illnesses reported in 2001 in the United States [2]. Still, occupational injuries tend to be an underestimated contributor to the burden of disease among U.S. workers [3,4].

Immigrant workers are a sizable proportion of the total U.S. workforce, and their numbers are growing [5,6]. In 2004, they were approximately 14.5% of the total workforce, and about 49% of the foreign-born were Hispanic [7]. In 2010, the foreign- born made up 15.8% of the workforce; Hispanics accounted for 49.9%, and Asians accounted for 21.8 percent of the foreign-born labor force [8]. Safety and occupational injury prevention have become an important issue among the U.S. immigrant population, in part, because a significant proportion of immigrant workers are believed to work in dangerous industrial and agricultural occupations [5,9,10]. The National Occupational Research Agenda suggests conducting surveillance of occupational safety data in special populations because workers with certain biologic, social, or economic characteristics may be at increased risk of occupational injuries and illnesses [2].

It has been reported that rates of nonfatal occupational injuries are actually significantly lower among immigrant workers compared to U.S.-born workers, but immigrant workers may suffer from more severe injuries than U.S.-born workers [11]. This finding is consistent with other studies that have found lower rates of overall injuries among immigrant adults and children [12,13]. Although explanations for the lower injury risk among immigrants have been posited [13], the underlying factors for this phenomenon are far from clear. One limitation of these previous studies is the cross-sectional study design of the survey data.

Many immigrant families either lack insurance or are not adequately covered by medical insurance [14,15]. Workers' compensation is a form of insurance providing wage replacement and medical benefits to employees injured from occupational injuries or illnesses. It is estimated that more than 130 million workers in the U.S. are covered by the workers’ compensation and the cost in 2007 was about $85 billion [16]. However, many experts have suggested that the workers’ compensation system is too complicated for immigrant workers to understand, and fear of repercussions may discourage immigrants from filing workers’ compensation claims after occupational injuries [17]. Lack of medical insurance or barriers to access workers’ compensation may make immigrant workers less likely than U.S.-born workers to seek professional medical treatment after occupational injuries.

Public policy debates in the U.S. about whether the nation should restrict or expand health care coverage for immigrants has prompted studies on health care access, quality, and costs among immigrants [18]. For the most part, these studies have focused on health care access and quality of medical care. A small number of studies have examined medical utilization and have reported that immigrants’ medical utilization and expenditures are lower than those of U.S.-born counterparts [18-20]. One common explanation given is that immigrants are generally healthier than the U.S.-born, so they have fewer costly chronic conditions. Others have reported that immigrants are less likely to have health insurance and subsequently use less health care than the U.S.-born [19,21].

Although previous research suggests that the immigrant population has a lower risk of injuries overall and a lower risk of occupational injury than the U.S.-born population, one major limitation of previous studies is their cross-sectional study design [11-13]. No prospective cohort study about occupational injuries among immigrant workers in the U.S. has been published. In addition, no national study has been done to investigate medical seeking behaviors and medical expenditures as a result of occupational injuries among immigrant workers. The Medical Expenditure Panel Survey (MEPS) follows a nationally representative sample of households and conducts five interviews about each family member’s health care use, insurance coverage, medical expenditures and sources of payments for medical conditions. Using the MEPS data, three aims of this paper are: 1) to confirm the previous findings that immigrant workers have a lower rate of nonfatal occupational injuries than U.S.-born workers, 2) to investigate whether immigrant workers are less likely than U.S.-born workers to seek professional medical treatment after occupational injuries, and 3) to test the study hypothesis that the proportion of medical expenditures paid by workers’ compensation for occupational injuries is smaller for immigrant workers than for U.S.-born workers.

## Methods

Our research used two data sources: the Medical Expenditure Panel Survey (MEPS) [22] and the National Health Interview Survey (NHIS) [23]. The sampling frame of MEPS is drawn from the participants in NHIS. Immigration information is not contained in MEPS, so we used the NHIS data to identify immigrant workers in MEPS. We pooled and linked data from the 2003–2007 NHIS and the 2004–2009 MEPS to construct a database to investigate medical expenditures for nonfatal occupational injuries in the U.S.

The MEPS is conducted annually and cosponsored by the Agency for Healthcare Research and Quality (AHRQ) and the National Center for Health Statistics (NCHS). It provides nationally representative estimates of health care use, insurance coverage, medical expenditures and sources of payment for the civilian non-institutionalized population. The MEPS has two major components: the household component (MEPS-HC) and the insurance component (MEPS­ IC). MEPS-HC obtains data from a nationally representative sample of households through an overlapping panel design in which new respondents are sampled and recruited from NHIS respondents each year and are interviewed 5 times over a 2.5-year period. Respondents are questioned about medical expenditures incurred in a 2 year reference period. This provides continuous and current estimates of health care expenditures at both the person and household level for each calendar year. An additional component of MEPS, the medical provider component (MPC) supplements and corroborates information received from the MEPS-HC component; the information from the MPC is incorporated into the MEPS-HC data [22]. MEPS-IC is an annual survey of employers that collects information on the employer's health insurance offerings.

In the MEPS, total expenditures were defined as the sum of payments paid for medical care services, including out-of-pocket payments, payments from private insurance, payments made by Medicare and Medicaid, or payment by workers' compensation, or other sources. Payments for over-the-counter medications and for alternative medicine (e.g., acupuncture or chiropractic care) are not included. The AHRQ applies imputation methods using available charge and payment data in either the MEPS Household Component or the MEPS Medical Provider Component to replace missing expenditure data [24].

### Human participant protection

The data were collected with the informed consent of the respondents of the NHIS, following procedures approved by the Institutional Review Board of the National Center for Health Statistics. The institutional review board of the Research Institute at Nationwide Children's Hospital approved secondary analysis of the data for our study.

### Terms and definitions

#### Immigration status and workers

To determine the immigration status of respondents, responses to the question "Where were you born?" in the NHIS were used. A respondent was categorized as being immigrant if the birthplace was outside the U.S. The NHIS categorized respondents born in U.S. territories as foreign-born because they may have a culture different from mainstream U.S. culture and because respondents in U.S. territories come from more than one "culture." Therefore, in our analysis U.S.-born workers included only those individuals born in one of the 50 states or the District of Columbia. Less than 1% of respondents of MEPS did not report their birthplace. Our analysis indicated that there was no significant difference in sociodemographic characteristics (gender, age, education level, family poverty level, and having no medical insurance) between respondents with birthplace and those without birthplace information.

**Figure 1:**
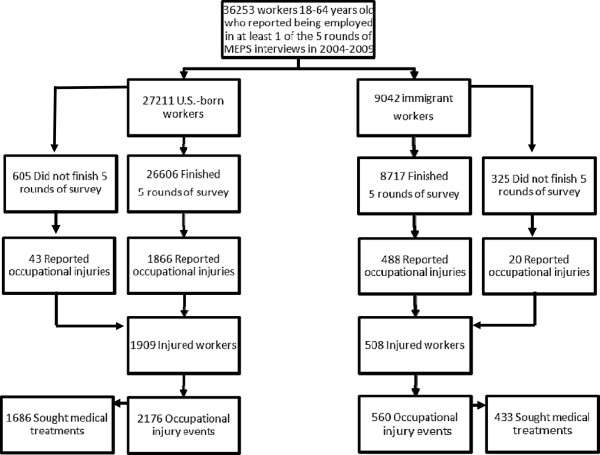
Eligible study participants.

Workers were defined as those who self-reported employment in any round of the *5* MEPS interviews. Some workers did not finish all five rounds so we calculated the number of follow-up days that these workers participated in the MEPS survey (Figure 1).

#### Occupational injuries

As described above, MEPS uses five rounds of interviews to collect detailed data on medical conditions, health care use, medical expenditures, sources of payment, and health insurance coverage for a 2 year reference period. When a medical condition is first reported in the MEPS, a portion of the MEPS Household Component questionnaire asks the respondent to specifically report whether this medical condition is an injury or not (yes or no). When an individual 16 years of age or older reports an injury in the MEPS, a number of questions are asked, including "Whether the injury occurred at work." In our study, injuries were defined as occupational injuries if the respondent said that the injury occurred at work. A respondent could have reported multiple injury events but each injury event has its own date of injury and event identification number.

In the 2004–2009 MEPS datasets, we found that some injuries occurred many years prior to the MEPS interview, but still had associated medical expenditures. Because our study aimed to investigate the medical expenditures of acute injuries that occurred during the MEPS reference period, we excluded a total of 140 injuries that occurred prior to the first MEPS interview. In this study, if no medical services were reported, we assumed medical care was not sought for that particular injury.

#### Health insurance coverage

The MEPS-IC collects data on health insurance plans obtained through private and public-sector employers. Data are obtained from employers through a prescreening telephone interview, a mailed questionnaire, and a telephone follow-up of non-respondents. In this article, we used the summary health insurance coverage variable in the MEPS for each respondent to categorize respondents as having private insurance, public insurance, or no insurance. The private insurance category includes respondents who, at any time in the survey year, had individual or group plan coverage for medical or related expenses or who were covered by TRICARE, the Department of Defense heath care program. Public insurance includes respondents who were never covered by private insurance or TRICARE during the year but who were covered at any time by Medicare, Medicaid, SCHIP (State Children's Health Insurance Program), or other state and local medical assistance programs. Those in the no insurance coverage category include respondents who did not have private or public insurance coverage at any time in the calendar year. This health insurance classification was used by the AHRQ to describe health insurance status of full-time U.S. workers in a recent report [25].

#### Statistical analyses

Data analyses were conducted using SAS [26]. All medical expenditures were adjusted to 2009 equivalent dollars using the Consumer Price Index for Medical Services published by the U.S. Bureau of Labor Statistics [27]. Due to the fact that multiple years of MEPS data were pooled together, we adjusted the final weight variable before our statistical analyses. AHRQ has recommended adjusting the analytic weight variable by dividing it by the number of years being pooled. This adjustment would have no effect on estimated means, proportions, or regression coefficients because the weight variable was being divided by a constant (i.e., number of years).

We first identified the total number of workers who reported occupational injuries, the total number of occupational injuries, and the total number of occupational injuries for which medical services were sought (those injuries with non-zero medical expenditures) in the MEPS sample. Using the survey design variables and the adjusted final weighting variable, we provide national estimates of the total number of injured workers, the total number of occupational injuries, and the total number of occupational injuries for which medical services were sought during the 2 year reference period. Using the follow-up days that each worker participated in the MEPS interviews and the total number of occupational injuries, we calculated an annual incidence rate and 95% confidence intervals (CI) of occupational injuries per 100 workers. We also calculated the proportion (%) and 95% confidence intervals (CI) of occupational injuries for which medical services were sought. We used the bootstrap methods with 1000 repeating bootstrap samples to calculate the 95% CIs [28].

Second, in order to test the hypothesis that immigrant workers were less likely than U.S.-born workers to seek medical services after occupational injuries, we used a hierarchical logistic regression modeling approach in which immigrant status and sociodemographic variables were treated as independent variables while seeking medical treatment was treated as the dependent variable. We included sociodemographic variables gender, age, race/ethnicity, marital status, education level, family poverty status based on federal poverty levels (FPL), and health insurance coverage of persons who reported occupational injuries. These variables are often adjusted as important factors in medical expenditures/costs studies [19,20,29].

Third, we provided national estimates of the average expenditures of occupational injuries according to the type of medical services and sources of payments and calculated the proportion (%) and 95% CIs of different types and sources of payment in the total expenditures during the 2-year MEPS reference period. Bootstrap methods were again used to calculate the 95% CIs. Results regarding the sources of payment allowed us to test the study hypothesis that the proportion of medical expenditures paid by workers’ compensation for occupational injuries is smaller for immigrant workers than for U.S.-born workers.

Finally, to estimate the average medical expenditures for occupational injuries per injured worker during the MEPS reference period and to assess the impact of above-mentioned sociodemographic variables on medical expenditures, we used multivariable linear regression models that have been used by others to analyze medical cost data [19,20,30]. Since the expenditure data were right skewed, a natural logarithm transformation was used to transform the expenditure data. Then we used linear regression to examine the association of the log-transformed expenditures with immigrant status, adjusting for sociodemographic variables. A smearing factor was applied to generate the final mean expenditures using Duan's approach to adjust for the impact of the log-transformation [31].

## Results

According to Figure 1, a total of 36,253 workers, 18–64 years old, were included in our study, including 27,211 U.S.-born workers and 9,042 immigrant workers. The majority of workers, 97.8% of U.S.-born workers and 96.4% of immigrant workers, finished all 5 rounds of the MEPS interviews. A total of 1,909 U.S.-born workers reported 2,176 occupational injury events and 508 immigrant workers reported 560 occupational injury events.

The national estimates of occupational injuries during the 2-year MEPS reference period were 10,422,717 for U.S.-born workers and 1,530,970 for immigrant workers between 2004 and 2009 (Table 1). Medical treatment was received for 77.3% (95% CI: 75.1%-79.4%) of occupational injuries suffered by injured U.S.-born workers and 75.6% (95% CI: 69.8%-80.7%) of the occupational injuries suffered by injured immigrant workers. However, the difference in the proportion of injuries for which medical treatment were sought was not statistically significant between two groups. The estimated annual rate of occupational injuries per 100 workers was 4.0% (95% CI: 3.8%-4.3%) for U.S.-born workers and 3.0% (95% CI: 2.6%-3.3%) for immigrant workers, suggesting that immigrant workers had a significantly lower rate of nonfatal occupational injuries than U.S.-born workers.

**Table 1:** Incidence of occupational injuries and the proportion of injured workers seeking treatment, MEPS Panels 9-23 (2004-2009)

	**MEPS Panel 9-13**				National estimates over the MEPS reference period (^*^numbers in millions)				Annual rate of occupational injuries per 100 workers^*^		**Sought medical treatment**	
	**n**	**Work injured persons**	**Work injuries**	**Sought treatment**	N^*^	Work injured persons^*^	Work injuries^*^	Sought treatment^*^	**%**	95%CI^**^	**%**	**95%CI**
U.S.-born workers	27211	1909	2176	1686	129.1	9.2	10.4	8.1	4.0	3.8-4.3	77.3	75.1-79.4
Immigrant workers	9042	508	560	433	25.9	1.4	1.5	1.2	3.0	2.6-3.3	75.6	69.8-80.7
Total	36253	2417	2736	2119	155.0	10.6	12.0	9.2	3.9	3.7-4.1	77.1	75.0-79.1

^*^Rates take into account the varying lengths of follow-up per worker. Workers (18-64 years old) were employed in at least one round.

^**^95% confidence intervals were calculated using bootstrap method with 1000 repeating bootstrap samples.

The unadjusted and adjusted odds ratios of seeking medical treatment after occupational injuries are presented in Table 2. In the unadjusted logistic regression model, immigrant workers appeared less likely than U.S.-born workers to seek medical treatment after occupational injuries (OR = 0.88, 95% CI: 0.63-1.21), but the difference was not statistically significant. In model 2 and subsequent models, female workers were significantly more likely than male workers to seek medical services after occupational injuries (OR = l.39, 95% CI: 1.07-1.80). In model 3, workers with middle or high income were more likely than those poor workers to seek medical treatment (OR = 1.61, 95% CI: 1.08-2.41 for middle income workers and OR = 1.61, 95% CI: 1.05-2.47 for high income workers). In the final model, gender and medical insurance coverage were the only two variables that significantly affected whether workers sought medical treatment after occupational injuries. Compared with workers with medical insurance coverage, uninsured workers were significantly less likely to seek medical treatment after occupational injuries (OR = 0.56, 95% CI: 0.42-0.75).

**Table 2:** Logistic regression analysis of seeking medical treatment after an occupational injury, MEPS Panels 9-13 (2004-2009)

	**OR**	**Model 1**	**OR**	Model 2^*^	**OR**	Model 3^*^	**OR**	Model 4^*^
		**95% CI**		**95% CI**		**95% CI**		**95% CI**
**Immigrant status**								
U.S.-born workers (ref.)	1.00		1.00		1.00		1.00	
Immigrant workers	0.88	0.63-1.21	0.91	0.61-1.36	0.92	0.61-1.39	0.98	0.65-1.47
**Gender**								
Male (ref.)			1.00		1.00		1.00	
Female			**1.39**	**1.07-1.80**	**1.43**	**1.09-1.86**	**1.34**	**1.03-1.75**
**Age (years)**								
18-24 (ref.)			1.00		1.00		1.00	
25-44			1.28	0.85-1.94	1.25	0.84-1.87	1.30	0.87-1.95
45-54			1.02	0.65-1.60	0.96	0.61-1.50	0.97	0.61-1.53
55-64			1.20	0.70-2.05	1.13	0.67-1.91	1.14	0.67-1.95
**Race/ethnicity**								
White, Non-Hispanic (ref.)			1.00		1.00		1.00	
Black, Non-Hispanic			0.99	0.69-1.42	1.02	0.71-1.46	1.01	0.70-1.44
Hispanic			0.85	0.59-1.24	0.88	0.60-1.29	0.91	0.61-1.35
Asian			1.20	0.47-3.03	1.15	0.46-2.92	1.07	0.42-2.74
**Marital status**								
Married (ref.)			1.00		1.00		1.00	
Widowed/divorced/separated			0.78	0.57-1.07	0.80	0.59-1.10	0.84	0.61-1.14
Never married/others			**0.70**	**0.52-0.95**	**0.71**	**0.53-0.96**	0.77	0.56-1.04
**Education**								
Less than high school (ref.)			1.00		1.00		1.00	
High school			1.08	0.79-1.49	1.03	0.75-1.42	0.95	0.69-1.32
College and higher			0.97	0.70-1.35	0.91	0.64-1.29	0.82	0.58-1.18
**Poverty status**								
Poor (<100% FPL) (ref.)					1.00		1.00	
Near poor (<100%-124% FPL)					1.48	0.80-2.74	1.47	0.80-2.70
Low income (125%-199% FPL)					1.34	0.86-2.11	1.27	0.81-1.98
Middle income (200-399% FPL)					**1.61**	**1.08-2.41**	1.40	0.93-2.12
High income (≥400% FPL)					**1.61**	**1.05-2.47**	1.33	0.84-2.09
**Medical insurance**								
Any private (ref.)							1.00	
Public insurance only							0.89	0.48-1.67
Uninsured							**0.56**	**0.42-0.75**

*For models 2, 3 and 4, the reported odds ratios (ORs) are adjusted for all other variables in the model.

Text is bolded when *P <* 0.05 from significance test of comparison with reference group.

*ref.* = reference group; *FPL* = Federal poverty level.

The estimated national expenditures for medical treatment of occupational injuries occurring during the 2-year MEPS reference period, by type of medical services and sources of payment are reported in Table 3. Based on the MEPS data, the estimated total expenditures during the MEPS follow up period were 20.70 billion (in 2009 U.S. dollars) during the study period. U.S.-born workers accounted for 18.45 billion (89.1%) and immigrant workers accounted for 2.25 billion (10.9%). The proportions of medical expenditures by type of medical service were comparable between U.S.-born workers and immigrant workers. For U.S.-born workers, 57.0% (95% CI: 49.4%-63.6%) of total medical expenditures of occupational injuries were paid by workers compensation, but for immigrant workers, 43.2% (95% CI: 33.0%-53.7%) were paid by workers' compensation (Table 3). Private insurance paid 30.3% of total medical expenditures for U.S.-born workers and 24.9% for immigrant workers. The percentage of total medical expenditures paid out-of-pocket was 6.7% (95% CI: 5.5%-8.3%) for U.S.-born workers and 7.1% (95% CI: 5.2%-9.6%) for immigrant workers, slightly but not significantly higher for immigrant workers. Other sources of payment were a larger proportion for immigrant workers (22.3%) than for U.S.-born workers (4.4%). Further analysis of this category revealed that for immigrant workers, over 80% of the ‘Other’ category was other types of private insurance including automobile, homeowner’s, liability, other miscellaneous/ unknown sources, and private insurance payments reported for persons not reported to have any private health insurance coverage (data not shown).

**Table 3:** Medical expenditures by type of services and payment sources for medical treatment of occupational injuries, MEPS Panels 9-13 (2004-2009)

	**U.S.-born workers**			**Immigrant workers**		
	**Total expenditures in the 2-year reference period (2009 U.S. $ in millions)**	**%**	95% CI*	**Total expenditures in the 2-year reference period (2009 U.S. $ in millions)**	**%**	**95% CI**
**Total expenditures**	18453	100.0		2251	100.0	
**By type of service**						
Ambulatory	11559	62.6	53.9-70.7	1348	59.9	48.4-71.7
Emergency department	2372	12.9	10.2-16.3	344	15.3	9.8-22.6
Hospital inpatient	4013	21.7	13.0-31.7	484	21.5	9.9-33.2
Home health	3	0.02	0.0-0.05	7	0.3	0.0-0.9
Prescribed medicines	506	2.7	2.2-3.4	68	3.0	2.1-4.2
**By source of payment**						
Family (out of pocket)	1241	6.7	5.5-8.3	160	7.1	5.2-9.6
Medicare	37	0.2	0.1-0.4	3	0.1	0.0-0.4
Medicaid	256	1.4	0.7-2.4	55	2.4	0.5-5.1
Private insurance	5595	30.3	24.6-36.8	560	24.9	17.3-33.9
Workers’ compensation	10514	57.0	49.4-63.6	972	43.2	33.0-53.7
Other sources^¥^	811	4.4	3.1-6.0	501	22.3	10.5-35.3

Note: *95% confidence intervals were calculated using the bootstrap methods with 2000 repeating bootstrap samples.

¥ Includes VETERANS, TRICARE, other federal, State/Local, and any other source not included above.

The mean expenditures per injured worker during the MEPS follow up period estimated by the multivariable linear regression models are reported in Table 4. Based on the 2004–2009 MEPS data, the mean medical expenditures per injured worker were comparable between U.S.-born and immigrant workers: $2357 for the U.S.-born workers and $2,351 for immigrant workers (*P* = 0.99). However, our results suggest that age, school education level, high income, and medical insurance coverage had statistically significant impacts on the mean medical expenditures after occupational injuries. Mean medical expenditures were significantly higher for older workers than for younger workers. Uninsured workers had significantly lower medical expenditures per injured workers ($1,885, *P* = 0.02) than workers who had private insurance ($2,557) and workers who had public insurance ($2,706).

**Table 4:** Linear regression analysis of medical expenditures o occupational injuries per injured worker during the 2 year MEPS reference period, MEPS Panels 9-13 (2004-2009)

	**U.S.-born workers**		**Immigrant workers**		**All workers**	
	Mean expenditures^*^(in 2009 U.S. $)	P^**^	Mean expenditures^*^(in 2009 U.S. $)	P^**^	Mean expenditures^*^(in 2009 U.S. $)	P^**^
**Immigrant status**						
U.S.-born workers (ref.)					2357	
Immigrant workers					2351	0.99
**Gender**						
Male (ref.)	2399		2265		2229	
Female	2772	0.19	1985	0.54	2486	0.26
**Age (years)**						
18-24 (ref.)	1892		915		1661	
25-44	2129	0.46	2436	**0.01**	1998	0.22
45-54	2843	**0.03**	3497	**0.00**	2706	**0.01**
55-64	3861	**0.00**	2593	**0.03**	3419	**0.00**
						**Race/ethnicity**						
White, Non-Hispanic (ref.)	2382		2980		2390		
Black, Non-Hispanic	2647	0.58	1172	0.06	2393	0.99	
Hispanic	2030	0.38	2337	0.39	2125	0.44	
Asian	3454	0.35	2476	0.61	2525	0.83	
**Marital status**							
Married (ref.)	2599		1648		2302		
Widowed/divorced/separated	2543	0.88	2249	0.23	2356	0.86	
Never married/others	2594	0.99	2571	0.17	2405	0.75	
**Education**							
Less than high school (ref.)	2932		1996		2646		
High school	2589	0.40	1987	0.99	2331	0.34	
College and higher	2258	0.07	2403	0.55	2114	0.08	
**Poverty status**							
Poor (<100% FPL) (ref.)	1971		1811		18200		
Near poor (100%-124% FPL)	2601	0.32	2141	0.72	2391	0.26	
Low income (125%-199% FPL)	2623	0.21	2464	0.38	2428	0.15	
Middle income (200-399% FPL)	2656	0.14	2319	0.46	2421	0.12	
High income (≥400% FPL)	3192	0.03	1933	0.86	2826	0.03	
**Medical insurance**							
Any private (ref.)	2867		1977		2557		
Public insurance only	2700	0.81	3571	0.07	2706	0.79	
Uninsured	2215	0.07	1350	0.12	1885	**0.02**	

^*^The original expenditure data were transformed using natural logarithm and linear regression models were fitted. Average mean expenditures were calculated using Duan’s smearing method.^29^

^**^P from significance test of comparison with reference group. *P* values< 0.05 are in bold text.

*ref.* = reference group; *FPL* = Federal poverty level.

## Discussion

We found that immigrant workers had a significantly lower incidence rate of nonfatal occupational injuries compared with U.S.-born workers. However, immigrant and U.S.-born workers had a similar likelihood of seeking medical treatment after occupational injuries. In addition, the estimated mean medical expenditures per injured worker during the 2-year MEPS reference period were comparable between the two groups, after controlling for gender, age, race/ethnicity, marital status, education, poverty level, and insurance status. Our results confirmed the third study hypothesis that proportion of medical expenditures paid by workers’ compensation for occupational injuries is smaller for immigrant workers than for U.S.-born workers (though this difference was only marginally significant).

The annual incidence rate of nonfatal occupational injuries per 100 workers in this study (3.9%; 95% CI: 3.7%-4.1%) matched the occupational injury and illness incidence rates reported by the Bureau of Labor Statistics for the 2004–2009 study period [1]. Furthermore, our results confirmed the previous findings that immigrant workers have a lower rate of nonfatal occupational injuries than U.S.-born workers [11-13]. Unlike previous studies based on cross-sectional survey data, this study used the longitudinal data from the MEPS. Longitudinal panel surveys have many strengths over cross-sectional surveys [32], therefore, this study adds to what is known about occupational injury risk among immigrant workers in the U.S.

There has been increased interest in recent years in understanding immigrant experiences with the health care system and their associated medical expenditures [33,34]. A recent systematic review of population-based studies of immigrants and their health care concluded that there is a dearth of information on medical expenditures among immigrants [29]. Of the 67 articles reviewed, 77% examined access to care, 27% studied quality of care, but only 6% examined medical expenditures [29]. Using the 1998 MEPS and 1996–1997 NHIS data, Mohanty et al. found that per capita total health expenditures among immigrants were 55% lower than those of U.S.-born individuals, and that immigrant children had 74% lower per capita health care expenditures than U.S.-born children [20]. As well, expenditures for uninsured and publicly insured immigrants were one half those for their U.S.-born counterparts [20]. Goldman et al. also found that immigrants, both documented and undocumented, had lower medical expenditures than their U.S.-born counterparts [35]. A study of health status and hospital utilization of recent immigrants to New York City found that immigrants were much less likely to be hospitalized for most major categories of illness, and they had lower mortality rates than U.S.- born persons [36]. Two factors have been suggested as the main reasons why immigrants have lower medical expenditures than U.S.-born individuals: immigrants are relatively healthier, and they may have less access to health insurance [19,35]. Welfare reform legislation, such as the Illegal Immigration Reform and Immigrant Responsibility Act, has substantially restricted recent immigrants’ eligibility for governmental health services [20].

Even less is known about the medical expenditures for occupational injuries among immigrant workers in the U.S. Results from this study suggested that immigrant workers were not less likely than U.S.-born workers to seek medical treatment after occupational injuries and that the average mean expenditures per injured workers during the 2 year MEPS reference period were about the same between the two groups. A recent study using 2002–2006 MEPS data to investigate medical care utilization for work-related injuries in the U.S. found that individuals with work injuries spend $1843 on average per year for treating work-related injuries [37]. The estimated mean expenditures in our study were higher due to the fact that we calculated mean expenditures per injured worker over a 2-year reference period and the expenditures were adjusted to be equivalent to 2009 U.S. dollars.

The proportion of medical expenditures paid out-of-pocket was slightly higher among immigrant workers compared to U.S.-born workers, but this difference was not statistically significant. This finding is consistent with previous studies that reported a slightly higher proportion of medical expenditures paid-out-of-pocket among immigrant adults in comparison with U.S.-born adults [19,35]. Another study of immigrant children’s medical care also found that Spanish speakers had 1.5 times the odds of spending $500 or more out-of-pocket medical expenditures per year than English speakers. Medical costs of occupational injuries and sources of payment were compared between Hispanic and non-Hispanic construction workers in the U.S., and it was found that Hispanic workers were less likely to receive workers’ compensation payment [38]. Results from our study also found that proportion of medical expenditures paid by workers’ compensation for occupational injuries was smaller for immigrant workers than for U.S.-born workers. Unlike a previous study that reported a significantly higher proportion of out-of-pocket payments in Hispanic construction workers than in Non-Hispanic White construction workers [38], our study did not find evidence of shifting medical expenditures to out-of-pocket payments. However, we did find evidence of immigrants’ greater use of other sources payment including automobile, homeowner’s, liability, and unknown sources of private insurance. More than 130 million workers in the U.S. are covered by the workers’ compensation [16] and, in theory, workers’ compensation could provide income benefits, medical payments and rehabilitation payments to injured workers and their families [39]. However, the regulations and the claiming process are possibly too complicated for immigrants to exercise their rights and to obtain benefits in the same way as U.S.-born workers [17,38]. Employers and workers’ compensation insurers can contest the workers’ compensation claims if they consider the injury is not work-related or because the worker wants more benefits than the employer and insurer are willing to pay [39]. A recent study from Quebec, Canada found that immigrant workers often need help from others to fill out their claim form, which usually are incomplete, or their claims are often contested by their employers [17]. A survey of unionized hotel room cleaners in Las Vegas reported that immigrant workers were less likely to file workers’ compensation, and, if they filed claims, their claims were more likely to be rejected than U.S.-born workers [40]. Administrative changes and education programs are needed to help immigrant workers to obtain the same benefits from workers’ compensation as U.S.-born workers after occupational injuries [38].

### Study limitations

Several limitations of our study should be mentioned. First, the NHIS and MEPS are government surveys in which undocumented immigrants who came to the U.S. illegally or who have overstayed their visas are likely to be underrepresented [41]. Accurate information on illegal citizens or citizenship was not available for our study. Second, immigrants in the U.S. are not a homogeneous group. Diversity in demographics, socioeconomic status, and culture exists across immigrant groups. The simplified classification of “immigrant” vs. “U.S.-born” would likely mask some of this diversity. Third, medical expenditure data from the self-reported survey of MEPS are subject to measurement errors and recall bias. It was not possible for us to test whether measurement errors and recall bias were different in immigrant workers as compared to U.S.-born workers. It has been estimated that aggregate national expenditures in the 2002 MEPS were about 13.8% below summary national expenditures from the National Health Expenditure Accounts [42]. Lastly, workers’ compensation programs are jointly managed by the federal and state governments, and research has suggested that the proportions of occupational injuries covered by workers’ compensation program differ significantly among the states [43-45].

## Conclusions

Unlike previous research, comparing overall costs of medical care between immigrants and the U.S.-born, that reported much lower medical expenditures among immigrants [19,20,35], results of our study suggest that U.S.-born and immigrant workers had a similar likelihood of have any medical expenditures for occupational injuries. Our research further indicates that the estimated medical expenditures for occupational injuries per injured worker were comparable between the two groups. Future research and government efforts are needed to reduce barriers to obtaining workers’ compensation benefits in immigrant workers and to promote safety using the workers’ compensation data [16,39].

## Abbreviations

AHRQ: Agency for Healthcare Research and Quality; CI: Confidence interval; FPL: Federal poverty level; MEPS: Medical Expenditure Panel Survey; MEPS-HC: Household component of MEPS; MEPS-IC: Insurance component of MEPS; MEPS-MPC: Medical provider component of MEPS; NHIS: National Health Interview Survey; OR: Odds ratio; SCHIP: State Children's Health Insurance Program; TRICARE: Department of Defense heath care program.

## Competing interests

The authors declare that they have no competing interests to report.

## Authors’ contributions

XH, JRW, and LB planned the study, and LB developed and refined the methodological approach. JS performed all statistical analysis and XH wrote the first draft paper. XH, KW, JS, WY discussed results and substantiated the interpretation of the results. KW, JRW, and GS helped in revising the paper. All authors read and approved the final manuscript.

## Pre-publication history

The pre-publication history for this paper can be accessed here:

http://www.biomedcentral.com/1471-2458/12/678/prepub
